# Increasing physical activity in postpartum multiethnic women in Hawaii: results from a pilot study

**DOI:** 10.1186/1472-6874-9-4

**Published:** 2009-03-03

**Authors:** Cheryl L Albright, Jason E Maddock, Claudio R Nigg

**Affiliations:** 1 Cancer Research Center of Hawaii, University of Hawaii at Manoa, Honolulu, Hawaii, USA; 2 Prevention and Control Program, Cancer Research Center of Hawaii, University of Hawaii at Manoa, Honolulu, Hawaii, USA; 3 Department of Public Health Sciences, John A. Burns School of Medicine, University of Hawaii at Manoa, Honolulu, Hawaii, USA

## Abstract

**Background:**

Mothers of an infant are much less likely to exercise regularly compared to other women. This study tested the efficacy of a brief tailored intervention to increase physical activity (PA) in women 3–12 months after childbirth. The study used a pretest-posttest design. Sedentary women (n = 20) were recruited from a parenting organization. Half the participants were ethnic minorities, mean age was 33 ± 3.8, infants' mean age was 6.9 ± 2.4 months, 50% were primiparas, and mean body mass index was 23.6 ± 4.2.

**Methods:**

The two-month intervention included telephone counseling, pedometers, referral to community PA resources, social support, email advice on PA/pedometer goals, and newsletters.

The primary outcome of the study was minutes per week of moderate and vigorous leisure-time physical activity measured by the Godin physical activity instrument.

**Results:**

All women (100%) returned for post-test measures; thus, paired t-tests were used for pre-post increase in minutes of moderate and vigorous leisure-time physical activity and comparisons of moderate and vigorous leisure-time physical activity increases among ethnic groups. At baseline participants' reported a mean of 3 ± 13.4 minutes per week moderate and vigorous leisure-time physical activity. At post-test this significantly increased to 85.5 ± 76.4 minutes per week of moderate and vigorous leisure-time physical activity (p < .001, Cohen's d = 2.2; effect size r = 0.7). There were no differences in pre to post increases in minutes of moderate and vigorous leisure-time physical activity among races.

**Conclusion:**

A telephone/email intervention tailored to meet the needs of postpartum women was effective in increasing physical activity levels. However, randomized trials comparing tailored telephone and email interventions to standard care and including long-term follow-up to determine maintenance of physical activity are warranted.

## Background

Young adult women, especially ethnic minority women, are at high risk for physical inactivity, or significant reductions in pre-pregnancy levels of physical activity (PA) following childbirthw. [1-4] Although guidelines indicate that most women can gradually resume or start PA 4–6 weeks postpartum [5], few women do. In a multiethnic sample of women, a significantly higher proportion (43.0%, p < 0.0003) of mothers were active before the pregnancy but inactive or irregularly active following childbirth; 21.5% were inactive before and after childbirth; 22.7% were active before and after childbirth; and 12.6% were inactive before but increased their PA after childbirth.[6] A study that followed primarily white women from early pregnancy to one year postpartum found similar percentages of women who reported rarely or never exercising one year postpartum.[3]

Few PA intervention studies have been tailored to postpartum women. However, a pilot study used stage-based materials, dervived from the Transtheoretical Model (TTM), along with four structured bi-weekly provider-delivered telephone contacts to increase PA in low-income women participating in the Women, Infants, and Children program (WIC). [7,8] The study sample consisted of largely of white (90%), married (66%), young (26.5 years), employed (70%) women. Results showed a significant increase in minutes of physical activity and energy expenditure after eight weeks of physical activity counseling.[7,8] Another exercise program to reduce post partum depression demonstrated improved fitness levels after 12-weeks of a walking program.[9] However, little has been done to improve PA in mothers of an infant less than 12 months of age, especially ethnic minority women.

Social Cognitive Theory (SCT) and TTM form the theoretical foundation for most physical activity interventions. Interventions using their constructs have been found to be effective with a wide-range of age groups, income levels, and ethnic groups.[7,10-15] SCT and TTM emphasize the development of behavioral skills by facilitating cognitive and behavioral processes via methods such as: stage-based materials, mediated support and counseling (via mail/telephone), goal setting, and skills-building classes.[11,15-23]

Prior to this pilot study, the authors conducted focus groups with 79 postpartum women to identify: 1) barriers to being physically active; 2) factors that facilitated a increase in physical activity; 3) exercise related resources or information that would help motivate women to become more active; and 4) postpartum women's receptivity to receiving SCT/TTM constructs such as telephone counseling revolving around short-term goals to increase their physical activity.[6] Based on the results from the focus groups the authors developed a SCT/TTM-based physical activity intervention, that included telephone counseling which was tailored to postpartum women. The purpose of this pilot study was to test the efficacy of this intervention to increase minutes per week of moderate to vigorous leisure-time physical activity (MVLPA) over two months.

## Methods

### Participants

Participants were recruited from Baby Hui, a non-profit organization that educates new mothers about infant care and parenting. A recruitment announcement was placed in Baby Hui's email listserve/newsletter. Inclusion criteria were sedentary (i.e., not doing more than 30 minutes a week of moderate or vigorous physical activity), 18–45 years of age, postpartum 3 months to 12 months, free of chronic diseases, not pregnant, and free of medical conditions that would limit moderate intensity PA.

Thirty-nine women were screened for eligibility. Five were ineligible at screening, and 20 (59%) were enrolled. Reasons for ineligibility included being too active (n = 2), infant was too old (n = 2), and unable to do aerobic exercise (n = 1). Fourteen of the screened women were not enrolled due to illness, scheduling problems, travel, or unidentified reasons.

Participants were enrolled at the Cancer Research Center of Hawaii's office, which is centrally located on the island of Oahu. The baseline and endpoint visits typically took between 30 and 40 minutes to complete and took place at the Center's office.

### Intervention Methods

A primary goal of this pilot study's intervention was to alter key mediators of physical activity including personal, social, and environmental factors that are integral to SCT/TTM theory. [14] These mediators have been the basis of previous research on exercise adherence and determinants; thus, there is empirical support for their efficacy.[14,24-27] Personal factors included cognitions, emotions, cultural values/social norms, and self-regulatory behavioral skills (e.g., self-monitoring, goal setting). Self-efficacy, operationalized for this study as one's confidence to be physically active in multiple situations, has been shown to be an important predictor of physical activity. [28-30] Therefore, the intervention was designed to enhance self-efficacy through promoting a series of successful experiences in meeting realistic physical activity goals. Other personal factors included enjoyment of physical activity, outcome expectations, benefits of physical activity, and skills related to overcoming barriers.[25] The intervention encouraged women to select enjoyable and practical activities, assisted them in developing solutions to barriers, and guided them in identifying personal benefits of an active lifestyle. Social factors included social support for initiating and maintaining physical activity from family and friends as well as peer support.[25] This was facilitated via tips on how to negotiate with their partner to provide facilitative or active support for physical activity.[27] Environmental factors included awareness of positive and negative environmental cues for MVLPA. Information about supportive environments were provided through referral to appropriate exercise facilities (e.g., health clubs with childcare services), tips for how to adjust physical activity for climatic changes (rain/hot weather), as well as information about community facilities and resources mothers could take their stroller (e.g., parks with paths for strollers). Teaching new mothers to apply self-monitoring, self-evaluation, and self-reinforcement through goal-setting, positive self-talk, and problem-solving was expected to develop skills that would enhance their ability to integrate physical activity into their daily lives. The physical activity intervention also focused on women at the contemplation/preparation and action stages of motivational readiness.[31,32] Table 1 lists the links between SCT/TTM theoretical mediators and the intervention methods. A two-month period was selected because previous studies have shown that 6–8 weeks of a personalized physical activity intervention that included counseling and goal setting was sufficient to facilitate initial changes in MVLPA. [7,8,19]

**Table 1:** Physical Activity Intervention Components

**Theory-Based Mediators of Behavior Change**	**Intervention Objective**	**Intervention Method**
**Personal variables**		
a. Self-efficacy	Setting and achieving a series of realistic short-term goals to produce multiple success experiences	Telephone counseling Problem solving
b. Barriers	Identify barriers to regular physical activity and develop ways to overcome them	Motivational interviewing Weekly Newsletters
c. Outcomes expectancy	Develop a realistic expectancies about the benefits of regular physical activity	Tip Sheets Email messages
d. Perceived benefits	Identify benefits of being active (e.g., more energy)	CDs and DVDs to dance with infant and other children
e. Enjoyable activities	Select enjoyable activities and do them in comfortable settings	Feedback about Pedometer steps
f. Self-monitoring/feedback	Record minutes of daily physical activity or pedometer steps; receive regular feedback on progress	

**Social factors**		
Support for physical activity	Guide women to ask family, friends, co-workers to assist with, or participate in, activities	Newsletters with tip sheets Telephone counseling
	Receive support & assistance from health educator	Tips on partner support

**Environmental factors**		
a. Access to facilities	Provide women with list of resources that list local physical activity facilities such as health clubs, walking & biking trails, stroller-friendly parks	Resource Directories
b. Access to resources that promote activity		Newsletters with resource information
c. Access to programs	Inform women about local activity-related events for families/women to be active	Telephone counseling Referral to physical activity websites

**Stages of change**		Newsletters
Use intervention methods appropriate to stage of change	Assess readiness to change or increase physical activities; provide relevant information or skills	Tip sheets Telephone counseling Emails

SCT/TTM methods were delivered during telephone counseling calls and were incorporated into print materials (tip sheets and newsletters) given to participants. At the baseline visit, participants had an individualized counseling session with the health educator. During this visit the health educator discussed the woman's personal benefits and barriers to physical activity, helped her problem solve her most significant barrier(s), negotiated her first physical activity goal, and set the time for her telephone calls. The telephone counseling was designed to provide regular, credible, individualized counseling. This was accomplished through the use of structured protocols (similar to a script) health educators were trained to follow and that have been shown to be effective in previous studies.[11,14,33,34] The telephone contacts set short-term physical activity goals in order to increase self-efficacy, evaluated success in meeting physical activity goals, problem solved social/environmental barriers to adherence, discussed future barriers and plans to effectively cope with them, and provided reinforcement and social support for physical activity (see Table 1). Specific information about physical activity or community resources were emailed afterwards, as appropriate. The schedule for telephone calls was once a week over the 2 month period. To encourage self-monitoring, women were given a pedometer (New Lifestyles NL-1000) a small, lightweight device that is clipped onto a belt or waistband and provides an objective measure of the number of steps accumulated each day. [35-37] Participants were instructed to write down their weekly minutes of activity and the number of daily steps on a calendar (provided to them at the baseline visit). During the first few minutes of each telephone counseling call, the health educator asked a woman how many steps she had accumulated each day over the previous week, then provided her with supportive feedback regarding attainment of her physical activity/daily steps goal, problem solved barriers to increasing the number of daily steps or minutes of physical activity, and helped the woman to set her next weekly goal.

Prior to recruiting any participants the first author (CLA) developed intervention materials tailored to new mothers and created structured guides telephone contacts that included checklists of items to be covered and scripts for how to address specific SCT/TTM constructs during a telephone call. She also developed detailed training manuals that highlighted common physical activity barriers and how to use motivational interviewing techniques to help a woman problem solve solutions to overcoming them. [38] The health educators used in this pilot study were two women who had a bachelor's degree and received instruction over a period of three months on how to follow the specific protocols for telephone- based physical activity counseling, and how to help new mothers set safe, realistic weekly incremental physical activity goals. The health educators were also trained to teach women behavioral skills that would help them become more active. The first author (CLA) has used similar methods to train the health educators from the three national sites that participated in the Activity Counseling Trial. [14]

Several strategies were used to monitor the health educators' adherence to counseling protocols, and to provide ongoing feedback and training, as necessary. For example, the health educators used an intervention tracking system (via an electronic spreadsheet) to document a woman's weekly physical activity goal, her progress in achieving her goals, the number of pedometer steps she accumulated each week, and her specific barriers to increasing physical activity. This physical activity data was reviewed during weekly staff meetings. Fidelity to the intervention protocols was monitored via the review of audiotapes of telephone calls and review of information sent to a woman via email.

### Data Collection

Participants completed a consent form, a questionnaire and had physiological assessments at baseline and 8–9 weeks later. The University of Hawaii's Human Subjects Committee approved study surveys and protocols. All of the participants provided endpoint data. A $30 gift certificate to a local shopping mall was provided at the end of the study.

### Physical Activity Measure

Respondents reported the number of days per week and minutes per day of moderate and vigorous leisure-time physical activity (MVLPA) at three intensity levels: 1) strenuous or vigorous activity (i.e., makes your heart beat quickly, makes you sweat); 2) moderate activity (i.e., doesn't make you tired, but makes you sweat a little); or 3) mild activity (i.e., takes little effort and doesn't make you sweat).[39,40] Reliability and validity of this measure is comparable to nine other self-report measures of exercise.[41] Minutes per week of moderate and vigorous activity were summed to create total minutes per week of MVLPA at baseline and endpoint.

### Barriers to Physical Activity

Respondents were asked to rate how much certain factors or psychosocial issues prevented or stopped them from being physically active. Each issue or barrier was rated on a likert style scale ranging from 1 to 5, with 1 = never, 3 = sometimes and 5 = very often (i.e., a higher score means the barrier frequently prevents the woman from being active). The barriers included feeling "self-conscious about her looks", "parenting duties" "not having time", "being too tired", "having no energy", "bad weather", and having "others discourage me".

### Body Mass Index

Weight was measured in light indoor clothing, without shoes, and height was measured, without shoes, using a portable stadiometer. BMI was calculated as the weight in kilograms divided by height in meters squared.

### Statistical Analyses

Paired t-tests were used to test for significant changes in continuous variables such as the increase in minutes of MVLPA from baseline to endpoint. Secondary t-test analyses included a comparison of change in MVLPA between races (e.g., white versus all other races) and between primiparous and multiparous women. The Cohen's "d" statistic was used to calculate an effect size, which is a measure of the magnitude of a treatment effect and is independent of sample size.

## Results

### Sample

Table 2 lists the sociodemographic characteristics of the sample. Sixty percent of the sample reported doing regular PA before they were pregnant. There were no baseline differences in demographic characteristics between whites and all other ethnic groups combined. Process data collected over the course of the intervention showed that on average of 5.8 ± 1.6 contacts out of the 7 scheduled contacts (83%) were completed. Over 90% of the mothers choose to walk during the intervention, all of them had strollers, and most (85%) had front-placed infant carriers (i.e., a "snuggly"). All of the participants received information about PA resources in their neighborhood, were emailed weekly newsletters with tip sheets addressing common barriers and benefits, and they set weekly goals based on minutes of MVLPA per week or daily accumulated steps on their pedometer (see Table 1). There was no significant change in BMI over the two-month period (note: the physical activity intervention was not designed to promote weight loss).

**Table 2:** Demographic Characteristics of sample

Sample size	20
Mean age (SD)	32.9 ± 3.8 years

Percent married	95%

Years of formal Education	16.8 ± 2.6 years

Percent working full or part time	45%

Ethnicity (percent)	
White	50%
Asian-American	35%
Native Hawaiian/other	15%

Mean age of infant (SD)	6.9 ± 2.4 months

Mean number Children (SD)	1.7 ± .8

Percent primipara	50%

Percent never smokers	65%

BMI (kg/m^2^)	23.6 ± 4.2

Mean number counselling contacts completed (SD) (total = 7)	5.8 ± 1.6

### Physical Activity Results

At baseline an average of 3 ± 13.4 minutes per week of MVLPA were reported and none met the recommended levels for weekly PA. This demonstrated their sedentary status at enrollment. After two-months of a tailored intervention the women's MVLPA increased to 85.5 ± 76.4 minutes per week (p < .001, Cohen's d = 2.2; effect size r = 0.7). The pre-post increases in minutes of MVLPA among ethnic groups, between women with an infant less than 6 months of age versus those with an infant older than six months, and between primiparous and multiparous women were all not significant. At endpoint 30% of the women met or exceeded national recommendations for 150 minutes per week of moderate or greater intensity physical activity. Over half (55%) had increases of 60 minutes or more of MVLPA per week.

### Barriers to Physical Activity Results

As noted in Table 1 the intervention specifically addressed postpartum women's barriers to becoming more physically active. The mean score across all 12 barriers was 2.57 (1.4) at baseline and the mean score at endpoint was 2.31 (1.2). This represented a significant (p < .03) pre-post reduction in perceived barriers. This pre-post change was not significantly different across ethnic groups or between primiparous and multiparous women.

### Feedback on intervention methods

At post-test participants provided anonymous feedback about the intervention. Over 75% found setting realistic weekly goals via the pedometer and receiving PA counseling from the health educators helped increase their PA. Over 80% were very satisfied with the amount of time they spent with the counselors, and 80% were satisfied with their PA progress during the intervention.

## Discussion

We successfully implemented a PA intervention to new mothers and their adherence to PA-related counseling contacts was high. Our tailored, home-based intervention was delivered though channels that are available and accessible to postpartum women: telephone and email. Postpartum women significantly increased MVLPA minutes over a two-month period. Our mean increase in minutes of MVLPA (82.5) was similar to change in minutes of PA reported in the WIC study (i.e., 88.2).[7] However, our results include minutes of moderate and vigorous PA while only moderate PA minutes were reported in the WIC study. Our sample had more race/ethnic diversity than the WIC study since half of our sample consisted of ethnic minority women who were also slightly older (by seven years) than the largely white WIC sample. [7] Finally, our study incorporated the internet (via email) as a method of contacting extremely busy postpartum participants, in addition to the telephone counseling sessions. Our participants were very receptive to our intervention methods and found them to be helpful. Our findings provide initial evidence that theoretically grounded approaches can be effective in enhancing behavior change in a population that faces unique challenges to becoming more active.

A limitation of this study is its use of self reported PA that was not objectively validated via accelerometers or other physiological assessments. Also, our PA assessment focused on leisure-time physical activities, and thus, did not include certain household activities that could achieve moderate intensity levels (e.g., vacuuming). Similar to the WIC study, our participants had one face-to-face contact in addition to the telephone contacts. Although these visits were required to obtain informed consent for a research study, our results cannot separate out the utility of this initial visit from the telephone/email counseling interactions. Thus, our results cannot demonstrate how effective a tailored PA intervention, delivered entirely over the telephone or internet, would be on women's MVLPA.

Although half our women were from an ethnic minority, our sample consisted of a convenience sample of educated, primarily married, sedentary but healthy, young women. Our sample, thus, consisted of women with a higher social economic status (SES) relative to the WIC mothers in the Moms on the Move pilot study. [7,8] Baby Hui members may respond differently to a tailored PA intervention compared to postpartum women from the same sociodemographic strata who are not members of a mother-baby support group. Thus, our results are only generalizable to similar populations of new mothers.

## Conclusion

A physical activity intervention, tailored to postpartum women, effectively increased participants' physical activity levels. However, a randomized trial comparing tailored telephone and email interventions to standard care and following women for long-term maintenance of PA is warranted.

## Competing interests

The authors declare that they have no competing interests.

## Authors' contributions

CLA conceived the study, its study design, coordinated the implementation of the intervention, and performed the statistical analysis. JEM and CRN participated in its design, the recruitment of subjects, and helped to draft the manuscript. All authors read and approved the final manuscript.

## Pre-publication history

The pre-publication history for this paper can be accessed here:


